# Image dataset of manalagi apple fruits for multi-class disease classification using deep learning

**DOI:** 10.1016/j.dib.2026.113092

**Published:** 2026-07-24

**Authors:** M. Aldiki Febriantono, Abba Suganda Girsang, Maria Susan Anggreainy, Suharjito

**Affiliations:** a School of Computer Science, Graduate Program, Bina Nusantara University, Jakarta 11480, Indonesia; b Computer Science Department, BINUS Graduate Program, Bina Nusantara University, Jakarta 11480, Indonesia; c Computer Science Department, BINUS Graduiate Program, Doctor of Computer Science, Bina Nusantara University, Jakarta 11480, Indonesia

**Keywords:** Apple fruit dataset, Agriculture, Disease identification, Deep learning, Computer vision

## Abstract

This dataset contains images of Manalagi apple diseases from Indonesia. Data collection was conducted from August 2024 to June 2026. Data were collected in apple orchards. All images were captured under natural environmental conditions. A total of 1168 unique Manalagi apple specimens were successfully documented. The specimens consisted of both healthy and diseased fruit. This dataset comprises four classes: Healthy, Anthracnose, Black Pox, and Powdery Mildew. Each specimen was observed and photographed directly. The documentation process yielded approximately 5100 raw images. The images were captured using various smartphone cameras and DSLR cameras. Each device has different camera specifications. The image size depends on the device used. Images that passed quality inspection were selected for the next stage. Each fruit specimen is cropped from the selected raw image. Each image was then labeled according to its disease class. The image size was standardized to 1024 × 1024 pixels. All images were saved in JPEG format. The curation process yielded 482 images. Each image represents a distinct fruit specimen.

Specifications Table

**Table:** 

Subject	Computer Sciences
Specific subject area	Apple fruit image identification using deep learning
Type of data	JPG.
Data collection	Data collection was conducted from August 2024 to June 2026. Data were collected at several apple orchards in Malang Regency, Indonesia. The data acquisition process yielded approximately 5100 raw images. The images were captured using smartphone cameras and DSLR cameras. Raw images that pass quality inspection are selected. Each image is cropped to retain only a single fruit Specimens. Each fruit is labeled according to its disease class. The size of each image is standardized to 1024 × 1024 pixels. The curation process yielded 482 images. All images were grouped into four classes. These four classes consist of Healthy, Anthracnose, Black Pox, and Powdery Mildew.
Data source location	Data source location: Location: Private Apple Orchard Kunci, Malang Regency, East Java, Indonesia Latitude: 8.035534° S, Longitude: 112.802262° E Zone: East Java, Country: Indonesia Poncokusumo, Malang Regency, East Java, Indonesia Latitude: 8.042039 ° S, Longitude: 112.807205 ° E Zone: East Java, Country: Indonesia
Data accessibility	Repository name: Mendeley Data Data identification number: DOI: 10.17632/9zgkwwv9j8.6 Direct URL to data: https://data.mendeley.com/datasets/9zgkwwv9j8/6
Related research article	Febriantono, M. A., Cahyono, N. H., Marcellino, B., et al. (2025). Lightweight Deep Learning Model Optimization for Apple Disease Classification Application. In 2025 International Conference on Applied Artificial Intelligence, Data Engineering and Sciences (ICAIDES). IEEE. https://doi.org/10.1109/ICAIDES67265.2025.11404049 Febriantono, M. A., Isnan, M., Hassan, R. (2026). Comparative Analysis of Mobilenet Architectures for Apple Disease Classification. In 2026 International Seminar on Intelligent Business and Edge-Computing Research (ISIBER). IEEE. https://doi.org/10.1109/ISIBER68248.2026.11470048 Febriantono, M. A., Girsang, A. S., Anggreainy, M. S., & Suharjito. Apple Disease Classification Using CLAHE-Enhanced MobilenetV3 Small With CBAM. [under review]

## 1 Value of the Data

• This dataset contains 482 images. The images are derived from 1168 unique Manalagi apple specimens. Each image represents a single apple selected as a sample.
• This dataset consists of four classes. The dataset classes consist of Healthy, Anthracnose, Black Pox, and Powdery Mildew.
• This dataset presents images of apple diseases obtained under actual orchard conditions. This dataset provides images of apple diseases to complement existing datasets.
• This dataset can be used as reference data for the development of an apple disease classification model.
• This dataset can be utilized in research within the fields of artificial intelligence, deep learning, and computer vision.
• It can be utilized by researchers, educational institutions, agricultural organizations, government agencies, and technology developers as a data source for plant disease research, image analysis, and the development of smart agriculture applications.

## 2 Background

Apples (Malus domestica Borkh.) play an important role in the horticulture sector. Apple production faces various diseases caused by fungi and bacteria. The disease can reduce the quality of the apples. Disease can also reduce the market value and shorten the shelf life of the fruit. This condition can reduce apple farmers' income. Early diagnosis of diseases supports successful apple cultivation. Early diagnosis of the disease can reduce economic losses for farmers. Early disease detection is a crucial step in apple orchard management. Advances in artificial intelligence support the development of disease diagnosis systems. Model performance is influenced by the quality of the dataset. Large datasets help improve the model's capabilities. Data diversity supports the model's generalization capability. Good annotations aid the model training process. Data collected under field conditions makes the model better suited for real-world use [1].

Most public plant disease datasets focus on leaf images. PlantVillage [2] is one of the most widely used datasets in plant disease research. Public datasets for apple diseases remain limited. Most public datasets cover only a few types of apple diseases. The number of images in public datasets of apple fruit diseases remains relatively small. Study [3] utilizes an apple fruit disease dataset comprising four disease classes. The study indicates that a more comprehensive apple fruit disease dataset is still needed to support the development of more reliable models. Study [4] developed an apple disease dataset for neural network-based diagnosis. The dataset includes healthy apples as well as those affected by scab, bitter rot, and black rot. Study [4] also emphasizes the importance of datasets that are larger, more diverse, and collected in orchards under natural conditions. Study [5] introduces an apple dataset collected directly in the orchard. The dataset is used to develop a YOLOv5-based object detection model. Study [5] demonstrates that a dataset from field conditions supports the development of a more reliable model.

This study aims to address the limitations of existing apple disease datasets. This article presents a curated dataset of images showing Manalagi apple diseases. All data were collected from apple orchards in Malang Regency, East Java, a major apple producing region in Indonesia. This dataset includes healthy apples and apples affected by disease. The diseases in this dataset include Anthracnose, Black Pox, and Powdery Mildew. All images were captured directly in the apple orchard under natural conditions. Image acquisition was performed under varying lighting conditions. Each image features a different orchard background. The position of the fruit varies across the images. Some fruit were obscured by leaves or branches during image capture. Each image was taken from a different camera distance. All images undergo a quality inspection process. The cropping process is performed on the part of the fruit showing symptoms of the disease. Experts label each image according to its disease class. All images are standardized to a uniform size. This process is carried out to produce a consistent dataset.

This dataset complements the existing collection of agricultural image datasets. It focuses on apple fruit diseases in tropical regions. All data were collected directly in orchards under actual field conditions. This dataset can be used for research on apple disease image classification. It can be used to evaluate and compare the performance of various deep learning models. This dataset supports research in the field of precision agriculture. It can also be utilized for research on intelligent crop monitoring and computer vision. This dataset aids in the development of more reliable apple disease diagnosis systems. Table 1 shows that this dataset complements the existing apple disease datasets.

**Table 1:** Existing apple disease studies.

Dataset Name	Type	Number of Classes	Total Dataset
PlantVillage Dataset [2]	Apple Leaf	4 classes of apple leaf diseases (Apple scab, Black rot, Cedar apple rust, and Healthy)	4645 images
Apple Leaf Dataset [6]	Apple Leaf	4 classes of apple leaf diseases (Scab, Black Rot, Cedar Rust, Healthy)	3171 images
Apple Disease Detection Dataset [7]	Apple Fruit	4 classes of apple fruit diseases (Blotch, Rot, Scab, Healthy)	319 images
AppleScabLDs [8]	Apple Fruit	2 classes of apple fruit diseases (Healthy, Scab Disease)	297 images
Manalagi Apple Fruit Disease Dataset (This study) [9]	Apple Fruit	4 classes of apple fruit diseases (Healthy, Anthracnose, Black Pox, Powdery Mildew)	4863 images

## 3 Data Description

This dataset contains images of Manalagi apples, comprising both healthy and diseased fruit. The data were collected in apple orchards located in Poncokusumo District and Kunci Village, Malang Regency, Indonesia, between August 2024 and June 2026. All images were captured under natural environmental conditions. Image acquisition took place across various weather conditions sunny, cloudy, overcast, rainy, and light drizzle and during both morning and midday hours. Air temperatures during data collection ranged from 21.4 °C to 25.8 °C. Air humidity during data collection ranged from 58% to 86%. Some images still show the orchard environment at the time of data collection. The dataset repository consists of three groups of data. The first group contains raw images. The second group contains Curated Images. The third group contains augmented training images. The raw images folder contains 5100 images. The images were captured using a DSLR camera and several types of mobile phone cameras. The size of each image depends on the device used. Device specifications are presented in Table 2. Image acquisition conditions and parameters are presented in Table 3.

**Table 2:** Camera specifications.

ID	Product	Model	Megapixels	ImageSize	FileType
D1	NIKON	NIKON D3400	24	6000×4000	JPEG
D2	Apple	iPhone 13	12.2	4032×3024	JPEG
D3		iPhone XS	12.2	4032×3024	JPEG
D4	Xiaomi	Redmi Note 7	12	3000×4000	JPEG
D5		Redmi Note 8	48	8000×6000	JPEG
D6		M2004J19C	13	3120×4160	JPEG
D7		Redmi Note 9 Pro	16.1	4640×3472	JPEG
D8	Samsung	SM-A225F	9	2992×2992	JPEG
D9		Galaxy A55 5G	9.4	4080×2296	JPEG
D10	Vivo	V25e	15.9	3456×4608	JPEG

**Table 3:** Image acquisition conditions and environmental parameters.

Date	Weather	Time	Temp ( °C)	Humidity (%)	Camera Device	Orchard Location
26-Jul-24	Mainly Clear	Afternoon (13:00)	25.8	58	D1 (17.6%), D5 (1.8%), D6 (17.9%), D7 (1.5%), D10 (61.2%)	Kunci
	Partly Cloudy	Afternoon (14:00)	25.4	63	D5 (2.4%), D6 (31.0%), D10 (66.7%)	Kunci
	Mainly Clear	Afternoon (15:00)	25.1	64	D5 (8.8%), D6 (18.4%), D10 (72.8%)	Poncokusumo
		Afternoon (16:00)	24.3	68	D7 (39.3%), D10 (60.7%)	Poncokusumo
16-Nov-24	Rain	Afternoon (14:00)	24.5	79	D2 (100%)	Kunci
	Light Drizzle	Afternoon (15:00)	24.3	79	D2 (100%)	Kunci
23-Nov-24	Overcast	Morning (06:00)	21.6	76	D2 (54.6%), D3 (41.0%), D7 (4.4%)	Poncokusumo
		Morning (07:00)	22.3	86	D2 (47.2%), D3 (37.9%), D7 (14.9%)	Poncokusumo
11-Jan-25	Overcast	Morning (07:00)	22.5	77	D2 (60.6%), D9 (39.4%)	Kunci
		Morning (08:00)	23.1	77	D2 (24.4%), D9 (75.6%)	Kunci
19-Apr-25	Overcast	Morning (07:00)	21.4	78	D4 (75.8%), D7 (24.2%)	Poncokusumo
		Morning (08:00)	22.8	83	D7 (100%)	Poncokusumo
12-Jun-26	Light Drizzle	Morning (10:00)	24	71	D7 (100%)	Poncokusumo
	Moderate Drizzle	Noon (11:00)	23.9	76	D7 (100%)	Poncokusumo
20-Jun-26	Mainly Clear	Morning (07:00)	20.4	80	D7 (100%)	Poncokusumo
	Clear Sky	Morning (08:00)	21.8	83	D7 (100%)	Poncokusumo
	Mainly Clear	Morning (09:00)	23.1	80	D7 (100%)	Poncokusumo

This dataset was constructed from approximately 5100 raw images. It comprises 1168 unique Manalagi apple specimens. All specimens are categorized into four classes. These four classes consist of Healthy, Anthracnose, Black Spot, and Powdery Mildew. A total of 482 fruit specimens were selected following quality inspection and curation. Each fruit specimen is represented by a single image. Each image is labeled according to its disease class. The resolution of each image was standardized to 1024 × 1024 pixels. Table 4 presents the dataset distribution for each class. The table displays the number of raw images. The number of unique fruit specimens is also presented. The number of curated images is shown for each class. The range of fruit specimen IDs is also included. Table 5 displays representative images of each class. A brief description of the visual characteristics of each class is also presented.

**Table 4:** Distribution of apple images and specimens.

Class	Raw Images	Unique Fruit Specimens	Curated Images	Fruit Specimen ID Range
Anthracnose	2892	659	147	A001–A659
Black Pox	800	136	136	B001–B136
Healthy	560	272	84	C001–C272
Powdery Mildew	848	101	101	D001–D101
Total	5100	1168	482

**Table 5:** Brief overview of the dataset.

Class	Definition	Sample
Healthy	Apples are categorized as healthy if they do not show symptoms of disease [10]. The texture of the fruit feels dense and hard. The fruit has a weight appropriate for its size. The surface of the fruit shows no signs of softening. The fruit has no bruises on its surface. The fruit skin appears clean and smooth. The fruit's color corresponds to its stage of ripeness. The fruit's surface looks fresh. The skin has a natural sheen. The skin is free of black spots, cracks, or holes. The fruit is round and symmetrical. The skin shows no signs of shriveling. There is no mold growth on the surface.	
Anthracnose	Apple anthracnose is also known as bull's eye rot [11]. This disease is caused by a fungus of the genus Neofabraea. This fungus causes rot in apples. Early symptoms are characterized by the appearance of small brown spots. The spots enlarge. The lesions become round and depressed. The color of the lesion changes from dark brown to black. The lesions continue to expand as the infection progresses. The fruit tissue begins to dry out. The fruit surface becomes harder. The fruit appears shriveled in the advanced stages of the disease.	
Black Pox	Black pox disease in apples is caused by the fungus Helminthosporium papulosum [12]. This disease attacks the skin of the fruit. The initial symptoms consist of small, round lesions. The lesion appears slightly depressed. The diameter of the lesion ranges from 3 to 9 mm. The color of the lesion ranges from dark brown to glossy black.	
Powdery Mildew	Powdery mildew disease in apples is caused by the fungus Podosphaera leucotricha [13]. Early symptoms appear as a white, powdery coating on the surface of young tissues. Initial infection on young fruit is characterized by the appearance of white mycelium. Fruit skin development is stunted. The infected part of the fruit suffers from impaired growth.	

## 4 Experimental Design, Materials and Methods

**Fig. 1:**
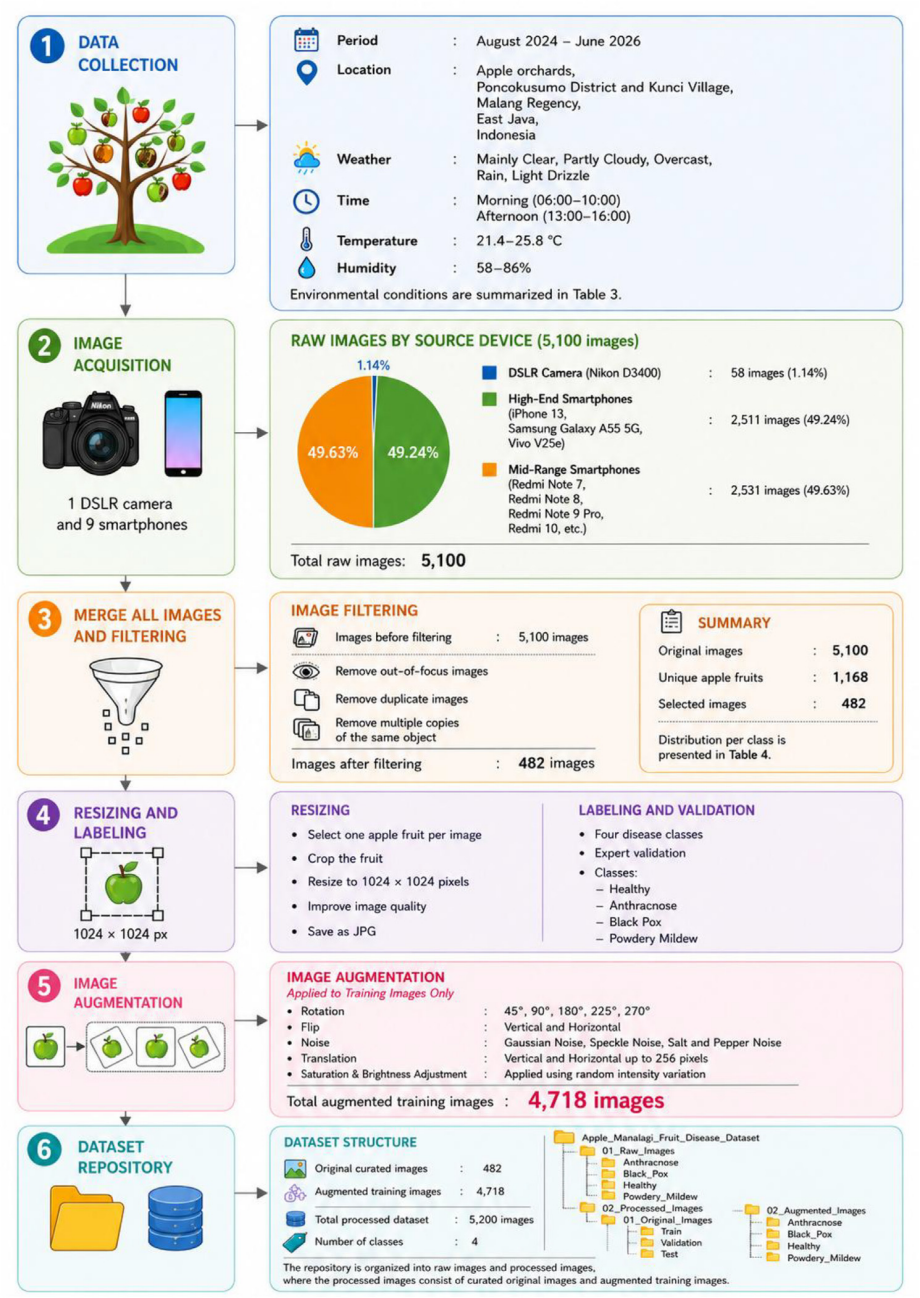
Workflow of the dataset collection process.

The development of this dataset was carried out in several stages. The first stage is data collection. The next stage is image acquisition. All images undergo quality inspection and preprocessing. The dataset is then divided into training, validation, and testing data. The training data is expanded through image augmentation. All data is then organized into a dataset repository. Each stage is carried out sequentially. The dataset preparation workflow is shown in Fig. 1.

### 4.1 Data collection

Data collection was conducted from August 2024 to June 2026. Data were collected at apple orchards in Poncokusumo District and Kunci Village, Malang Regency, Indonesia. The selected apples represent healthy and diseased conditions. Image acquisition was conducted under natural environmental conditions. Data were collected under various weather conditions. Weather conditions included clear, partly cloudy, overcast, rainy, and drizzly weatherImage acquisition was performed in the morning and afternoon. Air temperature during data collection ranged from 21.4 °C to 25.8 °C. Air humidity during data collection ranged from 58% to 86%. Image acquisition parameters are presented in Table 3.

### 4.2 Image acquisition

Image acquisition was performed using a single DSLR camera and several smartphones. The devices used have diverse camera specifications. The data acquisition process yielded 5100 raw images. Differences in devices result in varying image sizes. The specifications for each camera device are presented in Table 2.

### 4.3 Quality control and image filtering

All raw images are stored in a single repository. The acquisition process yielded approximately 5100 raw images. The raw images comprise 1168 unique specimens of Manalagi apples. Each raw image undergoes a visual quality inspection. Image sharpness is checked during the inspection process. Lighting conditions are also evaluated. Visual artifacts are examined in each image. The clarity of disease symptoms is also assessed. All these aspects serve as criteria for quality inspection. Blurred images were excluded from the dataset. Duplicate images were not included in the dataset. Images with poor lighting were removed from the dataset. Images that did not clearly show symptoms of the disease were excluded from the dataset. Raw images meeting quality standards are selected for processing. Individual fruit specimens were subsequently cropped from the filtered raw images. Not all documented fruit specimens were included in the final dataset. A total of 482 fruit specimens were selected following a curation process. Specimen selection was based on image quality. The clarity of disease symptoms was also a selection criterion. Each curated image represents a single fruit specimen. The final dataset consists of 482 images.

### 4.4 Image preprocessing

Individual fruit specimens were cropped from the filtered raw images. Each cropped image was centered on the selected fruit specimen. Each image was then standardized to 1024 × 1024 pixels with a 1:1 aspect ratio and saved in JPEG format. Each image was labeled based on identifications made by a plant disease expert. The labeling process was validated by an expert from the Regional Research and Innovation Agency (BRIDA) of Malang Regency. The preprocessing stage yielded 482 images categorized into four classes: Healthy, Anthracnose, Black Pox, and Powdery Mildew.

### 4.5 Dataset split

The curated dataset is divided into three subsets. These subsets consist of training, validation, and testing data. A split ratio of 70:15:15 is used. The augmentation process is performed after the dataset split. Augmentation is applied only to the training data. The dataset is split after the curation process. Each curated image represents a single fruit specimen. Each fruit specimen is assigned to only one subset. The training, validation, and testing datasets are mutually exclusive. This partitioning helps prevent data leakage.

### 4.6 Augmentation images

Augmentation was applied only to the training data. Augmentation techniques included rotation, flipping, translation, the addition of Gaussian noise, speckle noise, and salt-and-pepper noise, as well as adjustments to saturation and brightness. The augmentation process generated 4718 additional images. Examples of the results from each augmentation technique are presented in Table 6.

**Table 6:** Dataset augmentation process.

### 4.7 Repository organization

**Fig. 2:**
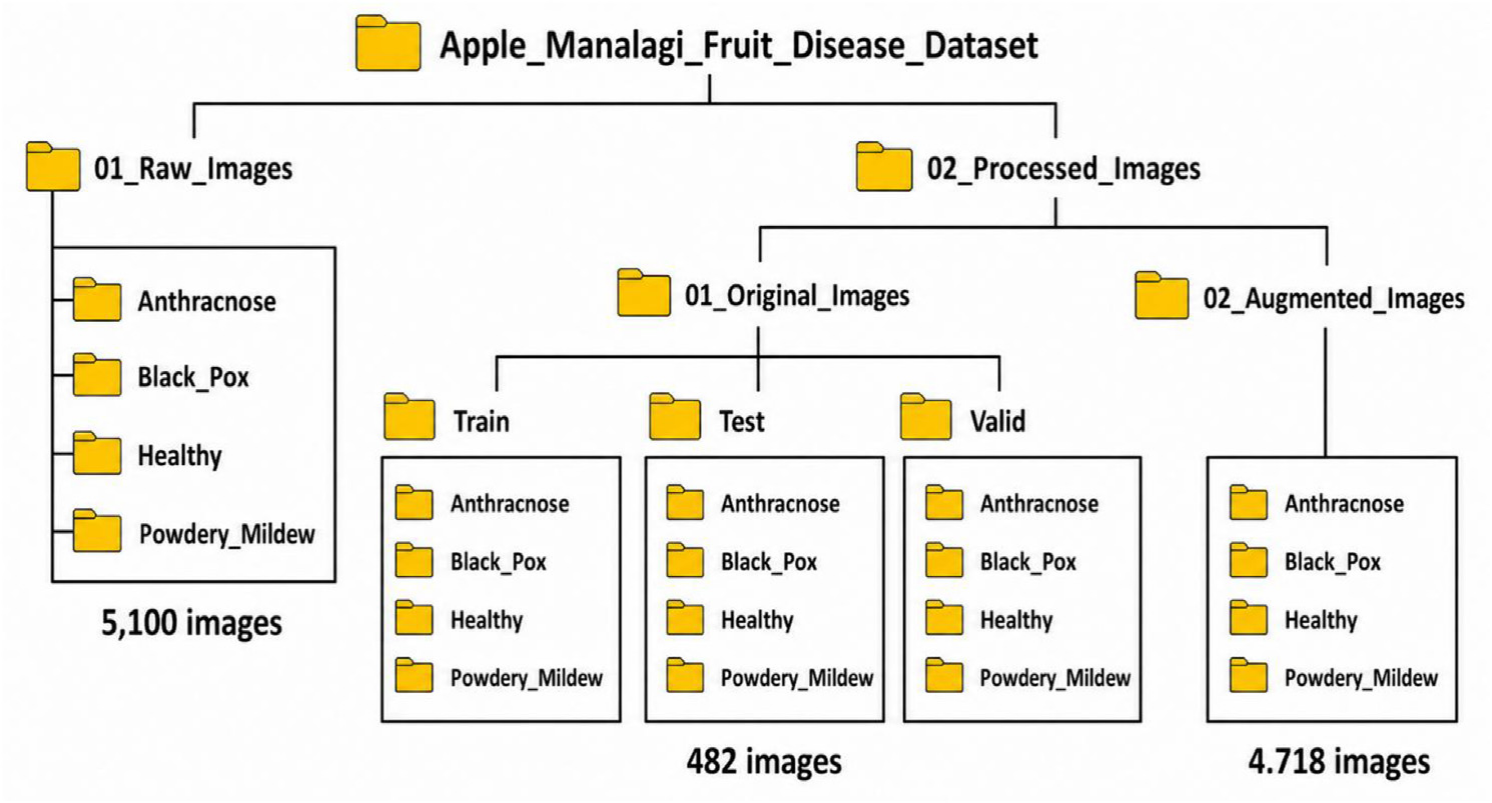
Dataset File Structure.

**Fig. 3:**
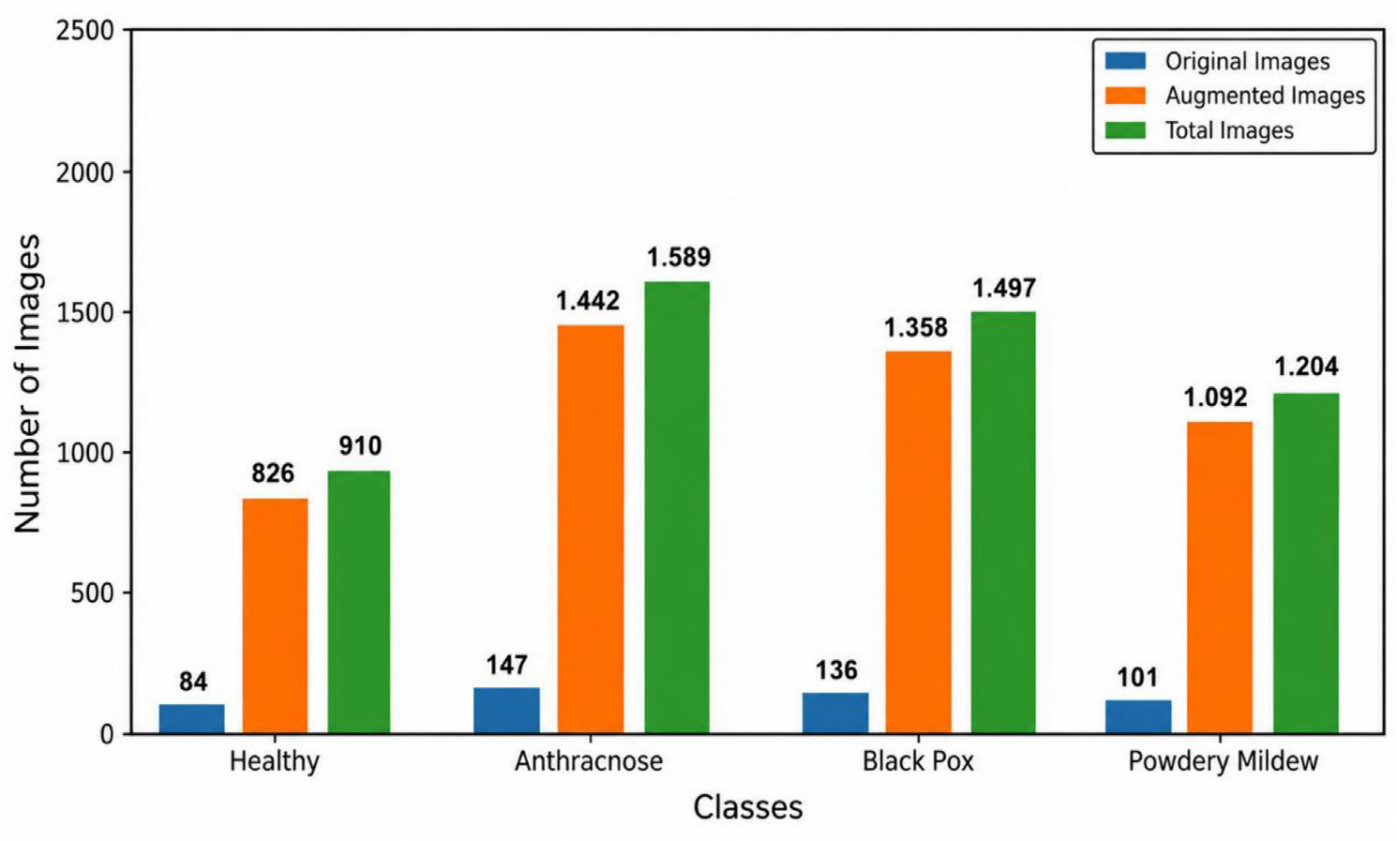
Image distribution in the dataset.

The dataset repository is organized into three primary folders: 01_Raw_Images, 02_Processed_Images, and 03_Augmented_Training_Images. The 01_Raw_Images folder contains 5100 original images collected during the image acquisition process. The 02_Processed_Images folder includes 482 curated images, which were subsequently divided into training, validation, and testing subsets using a 70:15:15 split ratio. The 03_Augmented_Training_Images folder comprises 4718 images generated by applying data augmentation techniques to the training subset. The complete dataset is organized into four disease categories: Healthy, Anthracnose, Black Pox, and Powdery Mildew. The overall organization of the dataset repository is illustrated in Fig. 2, whereas the distribution of images across the four categories is summarized in Table 7 and Fig. 3.

**Table 7:** Statistical overview of the apple dataset.

Class	Original Images	Train	Valid	Test	Augmented Images	Dimensions (pixels)
Healthy	84	59	12	13	826	1024 × 1024
Anthracnose	147	103	22	22	1442	1024 × 1024
Black Pox	136	97	21	21	1358	1024 × 1024
Powdery Mildew	101	78	11	12	1092	1024 × 1024
Total	482	337	72	73	4718	1024 × 1024

## Limitations

This dataset was collected from apple orchards in Malang Regency, Indonesia. The data were collected under various weather conditions. This dataset does not yet include environmental variations from other geographical regions. Environmental factors can influence the visual appearance of disease on apples. Differences in orchard conditions can lead to variations in image characteristics. Disease symptoms may appear different under varying environmental conditions. All images were captured under natural environmental conditions. The images were obtained using various smartphone cameras. Each smartphone has a different camera sensor. Sensor differences result in variations in image lighting. Sensor differences also affect image color. Image sharpness varies across devices. Image quality differs depending on the device used. This dataset contains only RGB images. Disease symptoms are observed based on the visual appearance of the fruit surface. This dataset does not provide multispectral images. Pathogen identification via laboratory analysis is not included. All labels have undergone a validation process by experts. Some disease classes exhibit similar visual symptoms. This symptom similarity can still lead to slight ambiguity during the annotation process.

## Ethics Statement

This study does not involve human participants. This study does not involve experimental animals. This dataset is publicly available. Its use must adhere to applicable citation guidelines.

## Credit Author Statement

**M. Aldiki Febriantono** contributed to conceptualization, visualization, validation, and writing original draft. **Abba Suganda Girsang** and **Maria Susan Anggreainy** contributed to methodology and data curation. **Suharjito** provided supervision and contributed to writing, review & editing.

## Data Availability

Mendeley DataManalagi Apple Disease Dataset (Original data)
